# Mutational Analysis of Highly Conserved Residues in the Phage PhiC31 Integrase Reveals Key Amino Acids Necessary for the DNA Recombination

**DOI:** 10.1371/journal.pone.0008863

**Published:** 2010-01-25

**Authors:** Shaohui Liu, Jinfang Ma, Wei Wang, Maoxiang Zhang, Qingting Xin, Siman Peng, Rongxiu Li, Huanzhang Zhu

**Affiliations:** 1 State Key Laboratory of Genetic Engineering, Institute of Genetics, School of Life Sciences, Fudan University, Shanghai, China; 2 MOE Key Laboratory of Microbial Metabolism, and School of Life Science and Biotechnology, Shanghai Jiao Tong University, Shanghai, China; University of British Columbia, Canada

## Abstract

**Background:**

Amino acid sequence alignment of phage phiC31 integrase with the serine recombinases family revealed highly conserved regions outside the catalytic domain. Until now, no system mutational or biochemical studies have been carried out to assess the roles of these conserved residues in the recombinaton of phiC31 integrase.

**Methodology/Principal Findings:**

To determine the functional roles of these conserved residues, a series of conserved residues were targeted by site-directed mutagenesis. Out of the 17 mutants, 11 mutants showed impaired or no recombination ability, as analyzed by recombination assay both *in vivo* and *in vitro*. Results of DNA binding activity assays showed that mutants (R18A, I141A, L143A,E153A, I432A and V571A) exhibited a great decrease in DNA binding affinity, and mutants (G182A/F183A, C374A, C376A/G377A, Y393A and V566A) had completely lost their ability to bind to the specific target DNA *attB* as compared with wild-type protein. Further analysis of mutants (R18A, I141A, L143A and E153A) synapse and cleavage showed that these mutants were blocked in recombination at the stage of strand cleavage.

**Conclusions/Significance:**

This data reveals that some of the highly conserved residues both in the N-terminus and C-terminus region of phiC31 integrase, play vital roles in the substrate binding and cleavage. The cysteine-rich motif and the C-tail val-rich region of phiC31 integrase may represent the major DNA binding domains of phiC31 integrase.

## Introduction

The integrase from the bacteriophage phiC31 was first described in 1991 as a 613 amino acid open reading frame recombinase [1]. It can precisely mediates site-specific DNA recombination between a bacterial attachment site (*attB*) and a phage attachment site (*attP*) without requiring others cofactors, resulting in integration when the two-*att* sites are present on two different DNA molecules and deletion or inversion when the two-*att* sites are on the same molecule [2], [3]. After recombination mediated integration, two hybrid attachment sites *attR* and *attL* are generated, which are themselves no longer target sites for phiC31 integrase. Therefore, unlike tyrosine recombinases, such as Cre and Flp [4], phiC31 integrase is a unidirectional integrase that only supports integration, and the resulting integration is stable. Presently, phiC31 integrase can perform recombination between minimal 34-bp *attB* and 39-bp *attP* sites in human cells [5] and mediates stable, site-specific integration of plasmids bearing *attB* into *attP* sites randomly integrated into the genomes of cultured human and mouse cells [6]. Furthermore, the phiC31 integrase recognizes native sequences in human and mouse genomes that possess partial sequence identity to *attP*, called pseudo *attP* sites, and mediates the integration of plasmids bearing an *attB* site into such pseudo *attP* sequences [6], [7]. This ability of phiC31 integrase to integrate into endogenous genomic sites has been used in gene therapy applications[8], [9], [10], [11] and engineering human embryonic stem cell lines and primordial germ cells [12], [13]. In addition, phiC31 integrase has been also used in the construction and manipulation of multiple model organisms, such as *Drosophila* [14], [15], [16], [17], *Xenopus* [18], and mice [19], [20]. These observations demonstrate that the phiC31 integrase is a valuable tool for gene therapy and genetic engineering.

The phiC31 integrase is a member of a serine-catalyzed superfamily of site-specific recombinases [21]. It belongs to the large serine integrase subfamily in which approximately 30 members share a similar modular organization of an N-terminal catalytic domain followed by an extended C-terminal region [21], [22]. The process of recombination by the large serine recombinases is thought to have many steps. The first step is recognition and binding of the substrates by the recombinase [23]. Protein–protein interactions between the recombinase subunits then bring the two substrates together in a synaptic complex [24]. Ghosh *et al*. showed that dimers of Bxb1 integrase bind the substrates *attB* and *attP*, which are then probably brought together to form a synaptic tetramer [25]. DNA cleavage occurs at a 2 bp crossover sequence site has been demonstrated in both phiC31 and Bxb1 integrases [26], [27], [28]. Strand exchange is thought to occur by 180° rotation of two recombinase subunits bound to half sites relative to the other two subunits [29], [30], [31]. Rejoining of the products is dependent on the complementarity of the DNA sequence at the staggered breaks; if there is a mismatch at this sequence, joining of the products is severely inhibited but iteration of strand exchange results in changes in the topology of the substrates [27], [32], [33].

Site mutagenesis and deletion analysis are usually applied in the study of protein functions. In three cases (phiC31 integrase, TnpX from the *Clostridium perfringens* transposon Tn4451 and TndX from the *Clostridium difficile* transposon Tn5397) site-directed mutagenesis of the proposed catalytic serine completely abolished their recombination activity [2], [34], [35]. In an attempt to screen for the phiC31 mutants that specifically recognized and integrated into target sites more efficiently, the technique of DNA shuffling was employed, and it had obtained many mutants, which with increased integration efficiency in human cells [36], [37]. Recently, we have shown that DAXX, an important cellular protein in human cells, can strongly bind to motif 451RFGK454 in the phiC31 integrase, resulting in the decrease of the integration efficiency of phiC31 integrase, indicating this region in the C-terminal domain of phiC31 integrase played an important role in protein-protein interactions [38]. Rowley *et al.* reported that a motif in the C-terminal domain of phiC31 integrase controlled the formation of the synaptic interface in both integration and excision, possibly through a direct role in protein–protein interactions [39]. They further demonstrated that substitutions in amino acid V129 in the N-terminal domain can lead to defects in synapsis and DNA cleavage, indicating that the N-terminal domain also has an important role in synapsis [40].

Conserved residues, which are usually believed to be the backbones of proteins, comprise pivotal structural and functional information accumulated during the long history of evolutionary screening. A sequence alignment of 30 serine recombinases revealed conserved regions outside the catalytic domain [21]. Until now, no system mutational or biochemical studies have been carried out to assess the roles of the conserved residues in the recombinaton of phiC31 integrase. Using site-directed mutagenesis, these residues were individually mutated to alanine. Several *in vivo* and *in vitro* assays were then used to investigate which of these residues are important for the recombination process and, furthermore, which steps of the recombination process are affected by each mutation. Our results show that mutation of some of highly conserved residues lead to a loss of biological activity and that these defects are due to impaired DNA binding affinity or specific target DNA cleavage, indicating that these conserved residues located both in the N-terminus and C-terminus region of phiC31 integrase are essential in DNA recombination. Multiple regions were found to bind substrate DNA, among them the cysteine-rich and the C-tail val-rich region may potentially be the major DNA binding domains of the integrase.

## Results

### Comparison of the Primary Sequence of Members in the Large Serine Integrase Family and Generation of PhiC31 Mutants

**Figure 1:**
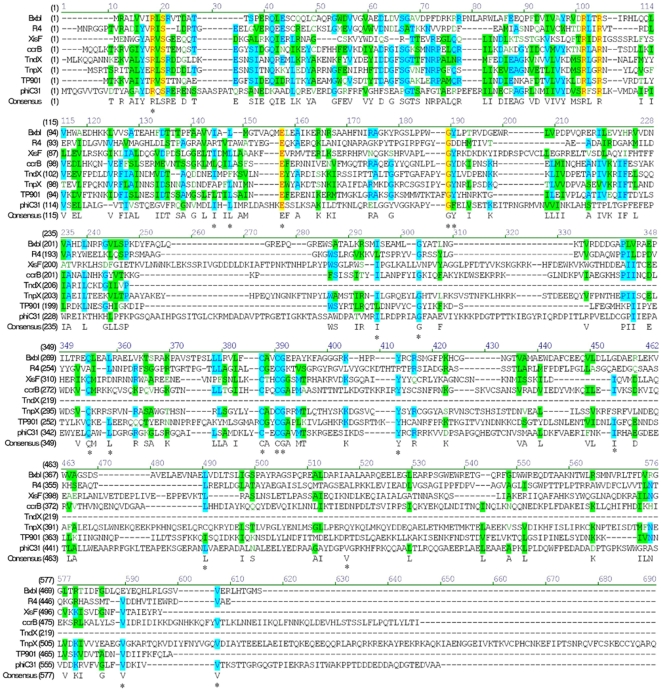
Sequence alignment of eight members including phiC31 integrase within large serine integrase family. The conserved residuals are shown in colour. Positions mutated in this study are indicated by asterisks. Sequence alignment was accomplished by using VNTI 9.0.

The phiC31 integrase has 613 residues and correspondingly a molecular mass of 68 kDa. By aligning its amino acid sequence with the serine recombinases family, we observed that it contains approximately 62 highly conserved amino acids residues (Fig. 1), and the cysteine-rich motif (aa372-403) followed by a leu/val/-rich region (aa 447–571) in the C-terminal domain of integrase. To determine the importance of conserved residues of phiC31 integrase during recombination, a series of 19 conserved residues were targeted by site-directed mutagenesis, generating 17 mutants (Table 1). To analyze the biochemical characterization of these mutants, the wild-type and mutant phiC31 integrases were expressed and purified as described in experimental procedures. The final purity of fusion proteins was above 95%. The phiC31 integrase was pooled using salt gradient elution and analysed by SDS-PAGE.

**Table 1:** Targeted residues and DNA sequence of mutagenic primers of phiC31 integrase.

Residue a	Conservation (% identity)^b^	Mutant(s)	Complementary primers
Arg-18	100	R18A	GTGCTTACGAC**GCT**CAGTCGCGCGAG CTCGCGCGACTG**AGC**GTCGTAAGCAC
Ile-141	80	I141A	GTCATGGACCTG**GCT**CACCTGATTATG CATAATCAGGTG**AGC**CAGGTCCATGAC
Leu143	60	L143A	GACCTGATTCAC**GCG**ATTATGCGGCTCGACGCG CGCGTCGAGCCGCATAAT**CGC**GTGAATCAGGTC
Glu-153	80	E153A	GCGTCGCACAAA**GCA**TCTTCGCTGAAGTC GACTTCAGCGAAGA**TGC**TTTGTGCGACGC
Gly-182Phe-183	80	G182AF183A	GAAGGCGCCTTAC**GCCGCC**GAGCTTGTTTC GAAACAAGCTC**GGCGGC**GTAAGGCGCCTTC
Ile288	100	I288A	CGTTATGCGA**GCC**CTTCGGGACCCG CGGGTCCCGAAG**GGC**TCGCATAACG
Gly296	80	G296A	CCGCGTATTGCG**GCC**TTCGCCGCTG CAGCGGCGAA**GGC**CGCAATACGCGG
Gln-347	60	Q347A	TATGAGCTT**GCG**GCGTGGTTGGACGGCA TGCCGTCCAACCACGC**CGC**AAGCTCATA
Leu-350	60	L350A	ATGAGCTTCAGGCGTGG**GCG**GACGGCAG CTGCCGTC**CGC**CCACGCCTGAAGCTCAT
Cys-374	100	C374A	CATGGACAAGCTGTAC**GCC**GAGTGTGGCGCCGTC GACGGCGCCACACT**CGG**CGTACAGCTTGTCCATG
Cys-376Gly-377	100	C376AG377A	AAGCTGTACTGCGAG**GCTGCC**GCCGTCATG CATGACGGCGGCA**GCCTCG**CAGTACAGCTT
Tyr-393	100	Y393A	ATCAAGGACTCT**GCC**CGCTGCCGTCGC GCGACGGCAGCG**GGC**AGAGTCCTTGAT
Ile-432	60	I432A	CATCTTCAACAAG**GCC**AGGCACGCCG CGGCGTGCCT**GGC**CTTGTTGAAGATG
Leu-468	60	L468A	CGGGCGAAC**GCT**GTTGCGGAGCGCG CGCGCTCCGCAAC**AGC**GTTCGCCCG
Val-495	60	V495A	GTACGACGGACCC**GCT**GGCAGGAAGCAC GTGCTTCCTGCC**AGC**GGGTCCGTCGTAC
Val-566	80	V566A	GTCGGGCTCTTC**GCA**GACAAGATCG CGATCTTGTC**TGC**GAAGAGCCCGAC
Val-571	80	V571A	GACAAGATCGT**TGC**CACGAAGTCGAC GTCGACTTCGTG**GCA**ACGATCTTGTC

a Numbers refer to positions in the wild-type 613 amino acid protein.

b Relation to a collection of 30 serine recombinases (http://www.blackwellscience.com/products/journals/suppmat/mole/mole2891/mmi2891sm.htm).

Mutated bases in the oligonucleotides are shown in bold.

### Mutations at Conserved Residuals both in the N-Terminus and C-Terminus Affect the Recombinase Activity

**Figure 2:**
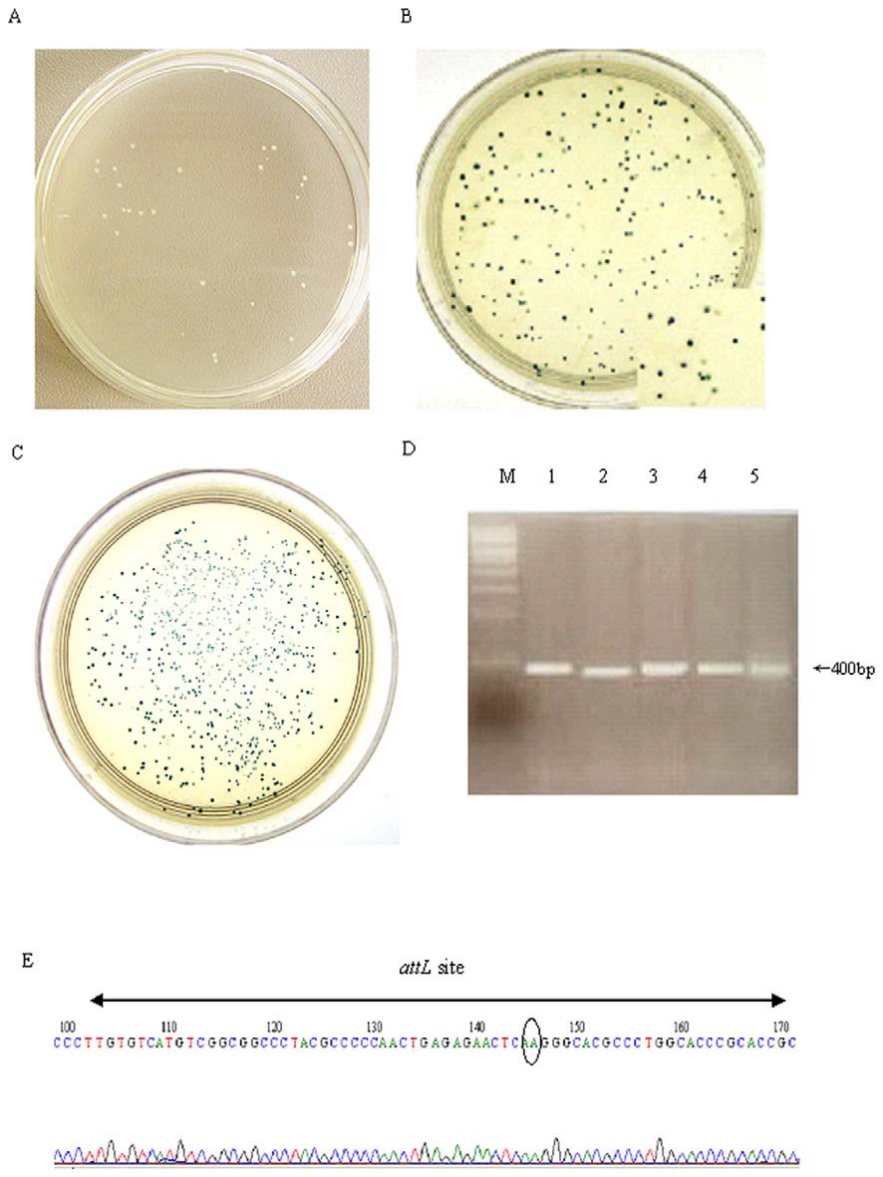
Recombination assay of phiC31 mutants in *E. coli*. Each mutant plasmid and its report plasmid were co-transformed into Top10 bacteria, respectively. The bacteria were then plated to form colonies on double antibiotic selecting agar plates containing X-Gal. When mutant recombinases still have their recombination ability, this recombination event consequently produces white colonies (A). When mutant integrase have lost or reduced recombination ability, It is observed that mixture of white and blue colonies (B) or only blue coloneies (C) on the plates. To further verify site-specific recombination at molecular level, the white colonies were picked, and plasmid DNA was subjected to PCR with specific primers that would amplify a 400-bp product only from the recombinant. DNA from 5 white colonies was subjected to PCR with specific primers, and a 400-bp product got amplified (D). The sequence of this PCR product contained the predicted chimeric *attL* site, comprising an *attP* arm (left) and *attB* arm (right) around the core AA dinucleotides (gray ellipse).

To test the recombination activity of mutant phiC31 integrases *in vivo*, the wild-type or mutant plasmid containing an ampicillin resistance gene, and the report plasmid pBCPB+ with *attP* and *attB* sites in direct orientation flanking a *lacZ* gene on a chloramphenicol resistant, were co-transformed into an *E. coli* strain Top10. The bacteria were then plated to form colonies on double antibiotic selecting agar plates containing X-Gal. As mutant recombinases still have their recombination ability, they will catalyze integration of *attP* into the *attB* site, resulting in the excision of the *lacZ* gene. This recombination event consequently produces white colonies. When mutant enzymes have lost their recombination ability, all colonies are blue. Our results showed that out of the 17 mutants, 6 mutants (I288A, G296A, Q347A, L350A, L468A and V495A) produced absolute white colonies as wild-type integrase (Fig. 2A), and the recombination frequency was nearly 100% as compared with wild-type protein (Table 2). Two mutants (I141A and I432A) produced blue and white mixed colonies (Fig. 2B), and the recombination frequency was 3.0%, 28.3% (Table 2), as compared with wild-type protein (*P*<0.05), respectively. Meanwhile, 9 mutants (R18A, L143A, E153A, G182A/F183A, C374A, C376A/G377A, Y393A, V566A and V571A) produced absolute blue colonies (Fig. 2C), and the recombination frequency was zero as compared with wild-type protein (Table 2).

**Table 2:** Summary and comparison of the results for band shift assays and the *in vivo* and *in vitro* recombination tests.

Mutants	Recombination activity		DNA binding affinities c
	*In vivo* (%)^a^	*In vitro* (%) b	*attB*
Wt phiC31	100	100	100
R18A	0	0	16±2.1
I141A	3.0±0.2	2.6±0.1	54±4.4
L143A	0	0	38±4.0
E153A	0	0	46±3.8
G182A/F183A	0	0	0
I288A	100	92.2±2.5	NA
G296A	100	96.1±3.1	NA
Q347A	100	98.6±2.5	NA
L350A	100	98.4±1.2	NA
C374A	0	0	0
C376A/G377A	0	0	0
Y393A	0	0	0
I432A	28.3±2.4	22.1±1.4	61±5.5
L468A	100	96.2±3.2	NA
V495A	100	95.2±3.8	NA
V566A	0	0	0
V571A	0	0	15.6±1.7

a Recombination frequencies *in vivo* = white colonies/total colonies×100%.

b Recombination frequencies *in vitro* = band densities of mutant with *attR*-L/band densities of wild-type integrase with *attR*-L×100%, assuming that 100% of the wild-type integrase in the reaction was active.

c The amount of free DNA substrate was plotted against concentrations of integrase to calculate the apparent binding affinities, Kd. NA, not applicable.

The values reported are mean±SD of at least three curves.

To further verify site-specific recombination at molecular level, the white colonies were picked, and plasmid DNA was subjected to PCR with specific primers that would amplify a 400-bp product only from the recombinant (Fig. 2D). The sequence of this PCR product contained the predicted chimeric *attL* site, comprising an *attP* arm and *attB* arm around the core AA dinucleotides (Fig. 2E), revealing that the recombination occurred between *attP* and *attB* was mediated by the phiC31 integrase.

**Figure 3:**
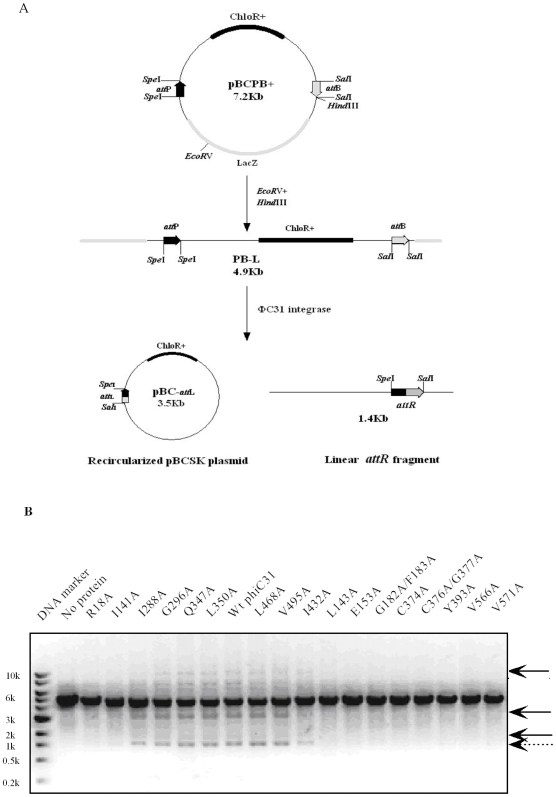
Recombination assay of the purified wild-type and mutant phiC31 integrases *in vitro*. (A) Schematic representation of functional phiC31 integrase assay. The linear 4.9 kb DNA substrate (PB-L) containing correct *attB* and *attP* sites was treated with wild-type or mutants phiC31 integrase, resulting in irreversibly produce a circular pBCSK molecule (or pBC-*attL*) and a 1.4 kb linear *attR* fragment (*attR*-L). (B) A 0.04 µM linear DNA substrate (PB-L) was treated with a 0.1 µM purified wild-type or mutant phiC31 integrase in recombination buffer at 30°C for 30 min and then stopped at 75°C for 10 min. The recombination reaction products were separated by electrophoresis on a 1% agarose gel. Arrows show the positions of circular molecule (solid line arrows), and a linear *attR* fragment (dotted line arrows). Wt is wild-type.

We carried out recombination assay of the purified mutant phiC31 integrase *in vitro*. A linear substrate PB-L containing correctly orientated *attB* and *attP* sites was employed for phiC31 integrase-mediated recombination reaction. When PB-L is catalyzed by phiC31 integrase, it will result in the excision of the DNA fragment between the *attP* and *attB* sites and be expected to give a recircular pBCSK DNA molecular with *attL* (pBC-*attL*) and a 1.4 Kb linear DNA fragment with *attR* (*attR*-L) (Fig. 3A). Thus, after recombination, besides a 4.9 kb band of linearized PB-L (incomplete recombination by phiC31 integrase), four other bands can be seen on agarose gel: a 1.4 kb band of linearized smaller recombination product, and three bands of the larger circular DNA molecule yielded by recombination (Fig. 3B). The linear *attR* fragment product could be directly separated on an agarose gel and stained with ethidium bromaide, and easily analyzed by densitometric measurement. As shown in Figure 3, more than 40% of the substrate (40 nM) was cleaved by 100 nM wild-type phiC31 integrase. Under the same conditions, 6 mutants (I288A, G296A, Q347A, L350A, L468A and V495A) retained the recombination activity nearly as high as wild-type phiC31 integrase as compared with wild-type protein (Fig. 3B), the cleaved product of mutants I141A and I432A were observed to show a weaker intensity of bands than that obtained with the wild-type integrase (Fig. 3B), while no cleaved product was detected from 9 mutants (R18A, L143A, E153A, G182A/F183A, C374A, C376A/G377A, Y393A, V566A and V571A) (Fig. 3B). Each mutant activity relative to that of the wild-type phiC31 integrase was calculated (Table 2).

### Reduced DNA Binding Affinity Correlates with Decrease or Loss of the Recombination Activity

**Figure 4:**
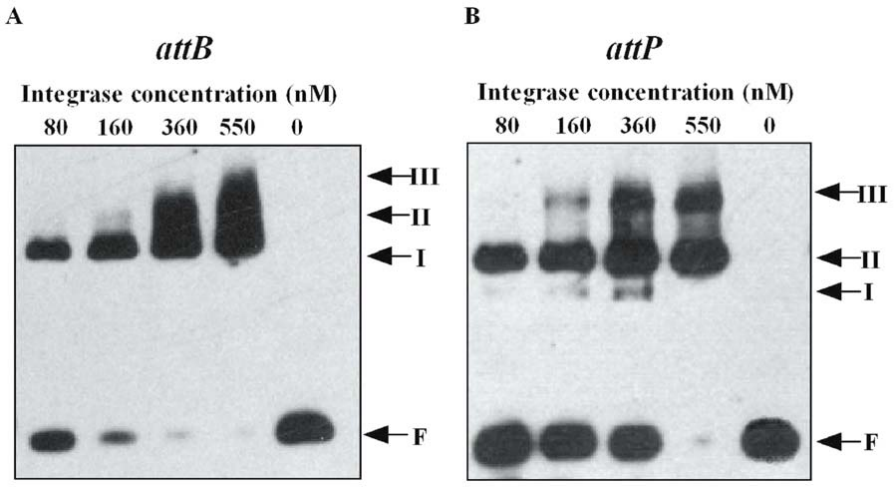
Assay of DNA binding activity of wild-type phi C31 integrase. A fixed amount (16 fmol) of a 41 bp biotin -labeled *attB* fragment (A) or a 50 bp biotin -labeled *attP* fragment(B) was incubated with varying amounts of purified wild -type phi C31 in binding buffer at 30°C for 30 min. Reactions were analyzed by polyacrylamide gel electrophoresis (5% polyacrylamide, 1x Tris-borate-EDTA). Arrows show the positions of free probe (F) and the DNA binding complexes (I, II and III).

**Figure 5:**
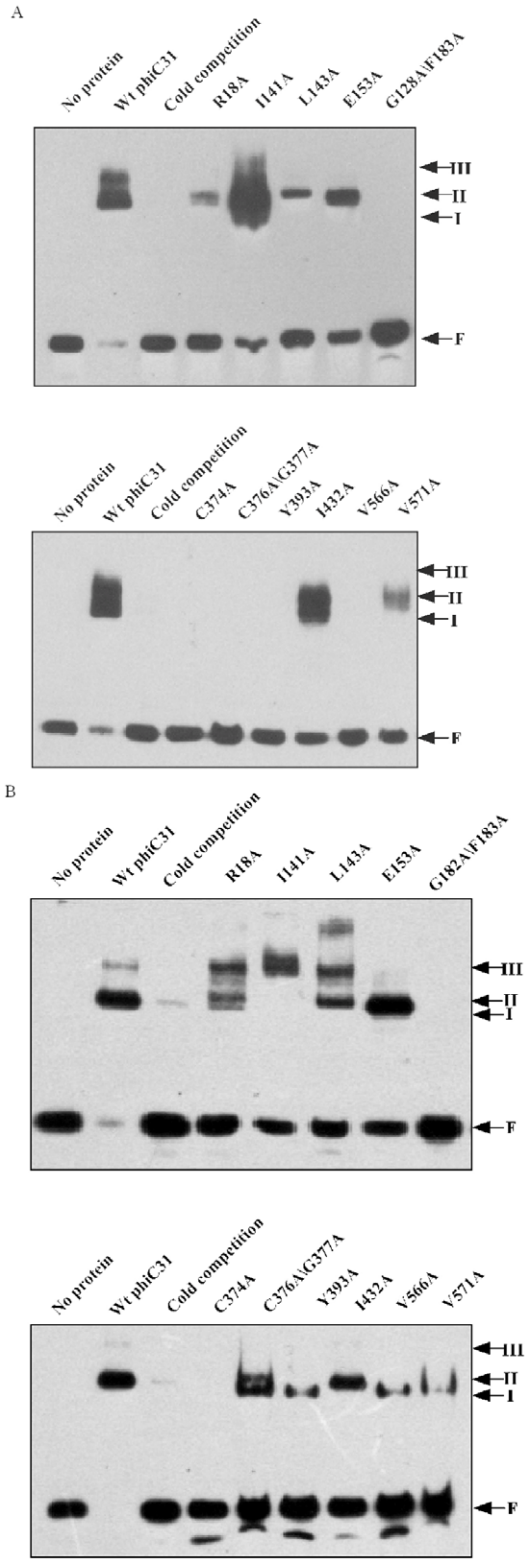
Assay of DNA binding activity of mutant phi C31 integrases. A fixed amount (16 fmol) of a 41 bp biotin -labeled *attB* fragment (A) or a 50 bp biotin -labeled *attP* fragment (B) was incubated with a similar amount of purified wild -type and mutant phi C31 in binding buffer at 30°C for 30 min. Results were analyzed by polyacrylamide gel electrophoresis (5% polyacrylamide, 1x Tris-borate-EDTA). Arrows show the positions of free probe (F) and the DNA binding complexes (I, II and III).

The recombination activity of the phiC31 integrase depends on its ability to recognize and bind specific DNA sites. To analyze the cause of decreased integrase activity of the mutants, the substrate binding activity was measured by electrophoretic mobility shift assay (EMSA). The characteristic of the complexes obtained by serial dilutions of wild-type phiC31 integrase binding to the attachment sites in bandshift experiments was considered at first. Adding increasing amount of wild-type phiC31 integrase to the reaction leads to the disappearance of the free-probe band, while three protein-DNA complexes of retarded electrophoretic mobility can be observed, referred as to I, II, and III, and the major species obtained corresponding to the complex II (Fig. 4A and 4B). These results are consistent with previous reports [26], [28], [41], [42]. To check if the observed shifted bands are specific, non-labeled *attB* probe was used as cold competition oligonucleotide, and was added in excess. The result showed that intense shifted bands of protein-DNA complexes disappeared in the presence of the cold competitor (Fig. 5A and 5B). Therefore, the shift observed was target specific.

To find out whether phiC31 mutants were still able to bind substrates, we analyzed their ability to generate protein-DNA complexes with *attB* (Fig. 5A). At the same concentration as wild-type protein(400 nM), we observed that mutants R18A, I141A, L143A, E153A, I432A and V571A were shown to have a great decrease in DNA binding affinity approximately 1–6 fold as compared with wild-type protein (*P*<0.05) (Table 2). Mutants I141A, I432A and the wild-type protein were shown to lead to the same three distinct complexes (I, II and III), while the band patterns of R18A, L143A and E153A were observed to include the formation of complexes (I and II) but with a weaker intensity of complexes than that obtained with the wild-type integrase (Fig. 5A). Especially, we observed that mutants G182A/F183A, C374A, C376A/G377A, Y393A and V566A had completely lost their ability to bind to the specific target DNA *attB* as compared with wild-type protein, as no complexes were seen in the gel retardation experiment (Fig. 5A).

In addition, we analyzed the ability of mutants to generate protein-DNA complexes with specific target DNA *attP*. At the same concentration of wild-type protein, we observed that mutants R18A, I141A, L143A, E153A, C376A/G377A, Y393A, I432A, V566A and V571A had an approximately 2–7 fold decrease in DNA binding affinity as compared with wild-type protein (*P*<0.05), and the band patterns of these mutants were different (Fig. 5 B). For example, mutants R18A and L143A generated protein -DNA complexes I, II and III, which was similar to that of the wild-type protein with *attP*; mutant I141A generated protein -DNA complexes III; mutants E153A and I432A generated protein-DNA complexes II; mutants Y393A,V566A and V571A generated protein -DNA complexes I; mutants C376A/G377A generated protein -DNA complexes I and II. Moreover, no complexes were seen in the gel retardation experiment with DNA *attP* for mutants G182A/F183A and C374A (Fig. 5B), indicating G182A/F183A and C374A had completely lost their ability to bind to the specific target DNA *attP*.

### Several Mutants with Lower DNA Binding Affinity Can Form a Synapsis but No Cleaved Intermediates

**Figure 6:**
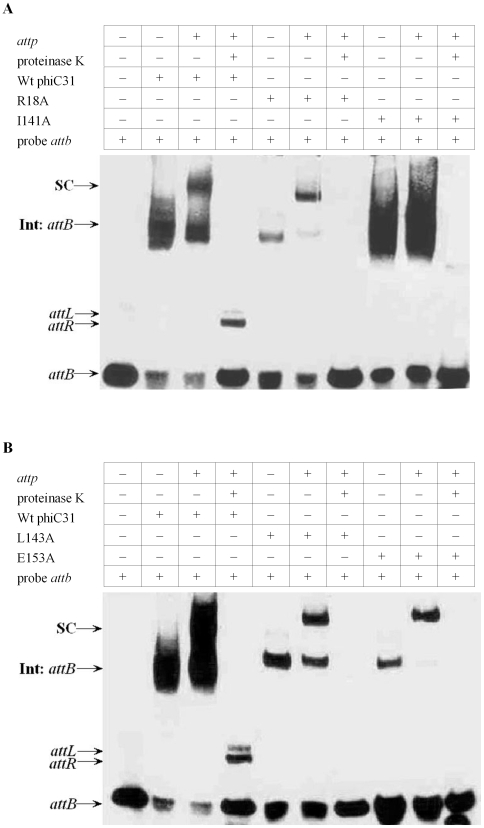
DNA synapsis and cleavage assays with wild-type and mutant integrases. Labelled *attB* was incubated with wild-type or mutant integrases (Panel A:R18A, I141A; Panel B:L143A, E153A; Panel C:I432A) in the presence of no cold *attP*, 198 bp cold *attP* in binding buffer and then untreated or treated with proteinase K. Reactions were analyzed by polyacrylamide gel electrophoresis (5% polyacrylamide, 1x Tris-borate-EDTA). The complexes observed are integrases (Int) bound to *attB* or synaptic complexes (SC). The recombinant products *attL* and *attR* are also indicated.

To investigate whether the mutants R18A, I141A, L143A, E153A and I432A that show the lower affinity for the *att* sites also affect the synapsis and cleavage steps, DNA synaptic complexes and cleavage ability of the proteins were measured by EMSA. The synaptic complexes and unbound substrates for the wild-type and mutants phiC31 were separated on the same gel to compare their synaptic complexes formation abilities at the same time. In binding assays with wild-type integrase in the presence of a labeled *attB* fragment and the 198 bp cold *attP* fragment, a major complex with the same mobility as the designated synaptic complexes was observed in addition to the shifted *attB* site (Fig. 6A). After proteinase K treatment, these complexes disappeared and the recombinant products *attR* and *attL* remains (Fig. 6A). Using the same conditions as in the wild-type integrase, we observed the band indicating the synaptic complexes in mutants R18A, I141A, L143A and E153A, but no bands showing the recombinant products, i.e. *attR* and *attL*, were detected (Fig. 6A and 6B). However, in mutant I432A, both of the synaptic complexes and the recombinant products *attR* and *attL* were detected, but the size of *attR* and *attL* have slightly difference as compared with wild-type protein (Fig. 6C).

### Mutants Completely Losing Their DNA Binding Affinity Have Structural Defects

**Figure 7:**
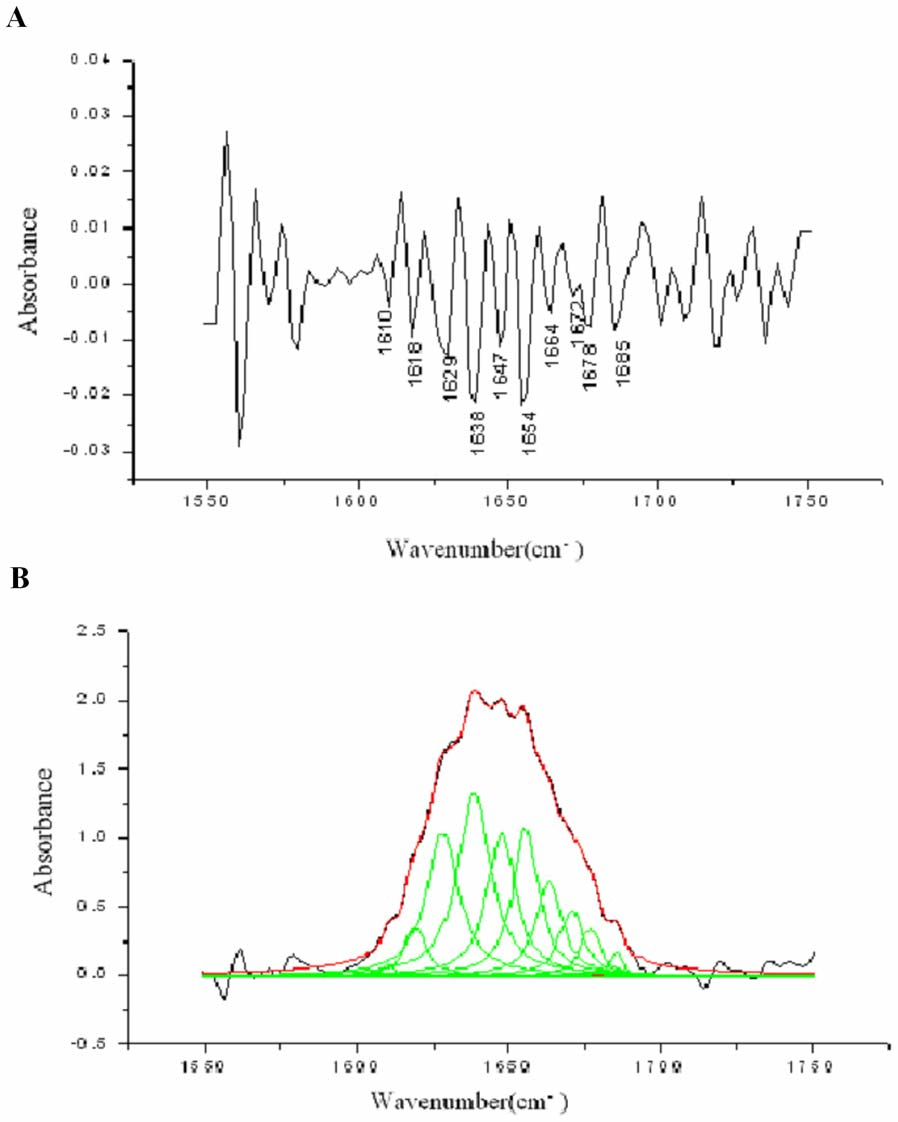
FT-IR spectra of the phiC31 integrase in heavy water. The second derivative of the infrared spectrum (A) and the absorption profile of the amide I' band (B).

**Figure 8:**
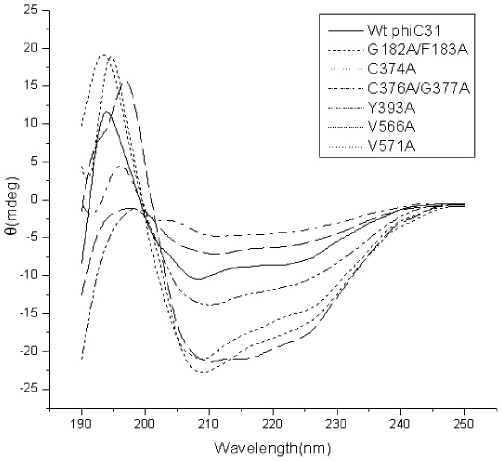
CD spectra of wild-type and mutant phiC31 proteins. Far UV spectra from 190 to 250 nm of wild-type phiC31 and mutants G182A/F183A, C374A, C376A/G377A, Y393A, V566A and V571A were shown, at 25°C using a 0.02-cm quartz cell. Protein was dissolved in 0.15 M sodium chloride, pH 7; Other conditions were described in Experimental Procedures. Wt is wild-type.

To determine if the secondary structure of these proteins had been significantly affected by the point mutations, we made an analysis on the secondary structure of the wild-type integrase by using fourier transform infrared (FT-IR) spectra. The result showed that wild-type integrase was composed of 15.2% α-helix, 41.5% β-sheets and 43.3% unordered structures (Fig. 7). We compared the circular dichroism (CD) spectra of the wild-type and mutant proteins losing DNA binding activities. The secondary-structure estimation, according to the CD spectra, revealed that wild-type integrase was composed of 17.1% α-helix, 42.6% β-sheets, 12.9% turns, and 27.4% random structures (Table 3), which is consistent with that of FT-IR spectra. Comparison to wild-type integrase CD spectra, mutants C374A and C376A/G377A did not result in significant changes in the protein secondary structure, however, mutants G182A/F183A, Y393A, V566A and V571A had a significant change at β-sheets and turn, respectively (Table 3 and Fig. 8), indicating that mutations of these residues were likely to affect proper folding of the β-strands into a hairpin structure.

**Table 3:** Secondary structure estimation from CD spectra of wild-type and mutant phiC31 integrases.

	α-helicity (%)	β-sheet (%)	Turn (%)	Random (%)
Wt phiC31	17.10	42.60	12.90	27.40
G182A/F183A	17.50	22.40	27.10	33.00
C374A	18.60	39.10	13.10	29.20
C376A/G377A	18.20	35.20	17.70	28.90
Y393A	17.70	22.30	27.20	32.90
V566A	23.80	24.90	22.30	29.00
V571A	23.90	26.90	16.40	32.80

## Discussion

Conserved residues are thought of folding nucleus of functional scaffolds in the history of protein evolution. They are also important clues in finding domain structures and further in protein engineering aimed to promote the applicable performance of selected enzymes [43], [44]. Sequence of phiC31 integrase can be aligned with other members of the serine recombinase family and it contains approximately 62 highly conserved amino acids residues. Our purpose is to find out if these conserved residues could give us more understanding of the domain architecture of the phiC31 integrase, which is still not fully identified. Recently, McEwan *et al* have found out that the large C terminal domain was necessary for DNA binding and synapsis [45]. Thus, previous research has shown evidence that both the N-terminus and C-terminus are crucially important in the recombination [39], [40]. However, we still cannot take a full picture of the process. So locating the DNA binding domain or those key residuals in substrate selection is currently a challenge in this field. Until now, no system mutational or biochemical studies have been carried out to assess the roles of the conserved residues in the integration process. In this study, we first employed site-directed mutagenesis to create 17 mutants, and verified the biological activity of these mutants *in vivo* and *in vitro*. As a result, It was found that mutants I288A, G296A, Q347A, L350A, L468A and V495A had a recombination activity as wild-type integrase, showing that these conserved residues were not important for recombination. However, mutants R18A, L143A, E153A, G182A/F183A, C374A, C376A/G377A, Y393A, V566A and V571A displayed no detectable recombination activity, and mutants I141A and I432A had a great decrease in activity, indicating that these conserved residues were essential for recombination activity.

A defect in the recombination activity may result from the inability of the mutant proteins to bind DNA substrates during recombination. Results of DNA binding activity assays showed that mutants (R18A, I141A, L143A, E153A, I432A and V571A) exhibited a great decrease in DNA binding affinity approximately 1–6 fold, and mutants (G182A/F183A, C374A, C376A/G377A, Y393A and V566A) had completely lost their ability to bind to the specific target DNA *attB* as compared with wild-type protein. These results indicated that these conserved residues play an important role in the protein-DNA interactions, their mutations had altered the ability of the mutant proteins to bind to the specific DNA targets, leading to the loss or decrease of their biological activity *in vivo*.

Sequence analysis showed that C-terminal domains of phiC31 integrase contained a cysteine-rich motif sharing a consensus LXCXCG21YXCX42C (aa 372–403), which could be a zinc finger-like domain, followed by a small leu/val-rich region (aa 447–571), which has a high probability of forming coiled-coil structures [21], [42], [46]. Like the phiC31 integrase, TpnX belongs to the large serine integrase subfamily, it also contained a putative zinc binding motif (residues 318–360) and a small leucine- and valine-rich region (aa 493–527). It has been shown that the dimerization domain of TnpX was localized to a region in close proximity to a putative C4 zinc finger domain and the leucine rich region [41], [42]. In addition, both of these motifs have been implicated in other proteins as dimerization domains that facilitate DNA binding [47]. Moreover, the C-terminal domains of Bxb1 and TnpX have been shown previously to be capable of binding specifically to DNA [25], [41], [42]. Our results showed that five mutations were located in the cysteine-rich motif and the Val-rich region in the C-terminal domain of phiC31 integrase: C374A, C376A/G377A and Y393A in cysteine-rich motif; V566A and V571A in the C-tail val-rich region. To our interest, mutations of five conserved residues located within these two region had almost completely lost their ability to bind to the specific target DNA *attB*. Therefore, we conclude that the cysteine-rich motif and the C-tail Val-rich region of phiC31 integrase may represent the major DNA binding domains of the phiC31 integrase.

We have previously demonstrated that cellular protein DAXX can strongly bind to motif 451RFGK454, which located within the leucine rich region in the C-terminal domain of phiC31 integrase, indicating this region played an important role in protein-protein interactions [38]. Rowley *et al.* reported that mutations in the C-terminal coiled-coil region cause hyperactivity, i.e., integrase is able to mediate both *attB* x *attP* and *attL* x *attR* synapsis and recombination, it indicats that this region has a direct role in protein-protein interactions in synapsis [39]. Together these data suggests that conserved residues located within a cysteine-rich motif and the leucine-and valine-rich region in the C-terminal domain of phiC31 integrase play an important role in both the protein-DNA and protein-protein interactions. However, further research is needed to identify if mutation of these conserved residues located within a cysteine-rich motif and the leucine-and valine-rich region in the C-terminal domain of phiC31 integrase will affect multimerization characteristic of these proteins.

What is more, the N-terminal conserved residuals are also involved in the DNA binding, such as, mutation of conserved residuals (G182A/F183) located within N-terminal domains of phiC31 integrase had completely lost their ability to bind to the specific target DNA *attB*. Therefore, we suggest that another putative DNA binding region also lies N-terminal of phiC31 integrase. Previous evidence demonstrated that there were multiple DNA binding regions located in N-terminus and C-terminus of other members of the large serine integrase family. For example, recent work on the serine integrase of mycobacteriophage Bxb1 suggests that at least two protein regions are required for efficient binding to its *att* sites [25]. The large serine recombinase, TnpX, have been also shown to have three DNA binding regions (aa 533–597, aa 243–261 and aa 598–707) [41], [42], [46].

Based on the results of mutants synapse and cleavage analysis, we have found more functional conserved residuals in the N-terminus of phiC31 integrase. Mutants R18A, I141A, L143A and E153A with lower affinities for the *att* sites can form a synapse with *attB- attP*, but the next step in recombination, i.e. cleavage and strand exchange is severely blocked. Similarly, in a previous study in which the affinity of the mutant S12A for wild-type *attP* and *attB* sites were assayed and found to be approximately 2-fold lower than the wild-type phiC31 integrase, and mutant S12A was able to form a synapse but unable to form the cleaved intermediates [28]. Rowley *et al* also showed that mutation of amino acid V129 in the N-terminal domain of phiC31 integrase could lead to defects in cleavage [40]. Together these data suggests that some conserved amino acids in the N-terminus of phiC31 integrase would also play such an important role in DNA cleavage.

Additionally, mutants I432A were shown to have a decrease in DNA binding affinity approximately 40% as compared with wild-type protein. This correlates well with our *in vivo* assay results, i.e. the recombination efficiency is proposed to be mainly affected by the decrease of binding affinity. So we further tested whether these mutants might be also defective in the step that follows DNA binding, i.e., synapsis and DNA cleavage. Our results showed that mutants I432A can form both the synaptic complexes and the cleaved intermediates, but size of *attR* and *attL* have slightly difference as compared with wild-type protein. The reason needs further studied.

In summary, our results show that mutation of some highly conserved residues lead to the loss of recombination activity and that these defects are due to impaired DNA binding affinity or affect specific target DNA cleavage, indicating that these conserved residues both in the N-terminus and C-terminus region of phiC31 integrase are essential in DNA recombination. We conclude that putative DNA binding regions lie in both the N-terminus and C-terminus of phiC31 integrase; especially, the cysteine-rich motif and the C-tail Val-rich region of phiC31 integrase may represent the major DNA binding domains of phiC31 integrase. Our work has also given clues for the further study of the N-terminus and C-terminus truncate mutation of phiC31 integrase to verify their roles in the DNA binding specifically and synaptic complexes formation. It offers useful information to those attempting to modify phiC31 integrase for more effective and more convenient applications in DNA manipulations.

## Materials and Methods

### Plasmid, Bacterial Strains and Culture Media

The plasmid pCMVInt, which expresses phiC31 integrase in mammalian cells under control of the cytomegalovirus (CMV) immediate-early promoter, and the pBCPB+ plasmid, which carries the phiC31 recognition site *attP* and *attB* in direct orientation flanking a *lacZ* gene on a chloramphenicol resistant *ColE1* derivative, were a kind gift from Dr. M.P. Calos. Restriction endonucleases, *pyrobest* polymerase and T4 DNA ligase, were purchased from TaRaKa (Dalian, China). The Ni-NTA agarose column, the expression vector pET22b+, and its host *Escherichia coli* BL21 (DE3) were obtained from Novagen (Darmstadt, Germany). Isopropyl-1-thio-β-D-galactopyranoside (IPTG), sodium dodecyl sulfate (SDS), dithiothreitol (DTT) and glycine were purchased from Sigma (St. Louis, USA). All reagents were of biotechnological grade.

### Recombinant DNA and PhiC31 Mutagenesis

To generate an expression vector for wild-type phiC31 integrase with C-terminal His-tagged, a 1.8 kb fragment was amplified from pCMVint by PCR with primers 5′- ATACATATGACACAAGGGGTTGTGAC-3′ and 5′-GTGCTCGAGGC CGCTACGTCTTCCGTG -3′. The amplified product, was digested with *Nde*1 and *Xho*1, and cloned into plasmid pET22b(+) digested with the same enzymes. The ligation mixture was used to transform *Escherich*ia coli strain and selected on LB-Agar plates containing ampicillin (100 µg/ml). The recombinant were analyzed by restriction digestion and confirmed by automated DNA sequencing (Invitrogen, shanghai, China), and named as pET22b-C31.

PhiC31 mutants obtained by using the QuikChange site-directed mutagenesis kit (Stratagene) according to the supplier's instructions, with plasmid pET22b-C31 as the template. For each point mutation, two complementary primers having mutated positions were used (Table 1). After initial denaturation at 95°C for 0.5 min, the cycling parameters were 0.5 min at 95°C followed by 1.0 min at 55°C and 12 min at 68°C (12 cycles). The reaction mixtures were placed on ice for 2 min, and then the parental, supercoiled double-stranded DNA was digested with 0.5 µl of *Dpn*I at 37°C for 1 h before being transformed into competent *E. coli* XL1 Blue cells. Mutations were verified by DNA sequencing, and named as pET22b-MC31.

### Protein Expression and Purification

The wild-type and mutant phiC31integrases expression plasmids were transformed into *Escherichia coli* strain BL21 (DE3), respectively. For expression, a single colony was used for inoculation in LB medium contained 50 µg/mL ampicillin. When the OD_600_ reached 0.8, the cells were induced by 0.1 mM IPTG for 30 h at 18°C with shaking. Then the cells were harvested and resuspended in cold lysis buffer H (20 mM Tris-Cl, pH 8, 500 mM NaCl, 20 mM imidazole). The lysate was disrupted by gentle sonication and the suspension was centrifuged for 40 min at 12,000 g. The lysate supernatant was directly loaded onto 5 ml Ni-NTA agarose column that was pre-equilibrated in lysis buffer H. After extensive washing in the same buffer, the column was eluted with lysis buffer H containing 500 mM imidazole concentrations. After SDS–PAGE, the pure fractions containing the recombinant protein were pooled and concentrated to 20 mg/ml in sample buffer (5 mM Tris-Cl, pH 8, 1 M NaCl) by ultrafiltration, then added 10% glycerol and stored at −70°C. Protein concentrations were determined by the Bradford assay.

### Recombination Assay *In Vitro*

Recombination assays *in vitro* were performed as described previously [28], with modifications. Briefly, the substrate for determining phiC31 activity was a 4.9 Kb linear DNA pBL from plasmid pBCPB+ digested with *Hin*dIII and *Eco*RV. When phiC31-mediated intramolecular recombination occurs between the *attP* and *attB* sites flanking an origin of replication and an antibiotic (chloramphenicol) resistance gene, the resulting products are two molecules. One product is a supercoiled pBCSK plasmid (pBC-attL) carrying *attL*, and the other is a single linear fragment *attR*-L carrying *attR*. All recombination reactions were in a 20 µl reaction volume containing enzyme reaction buffer (50 mM glycine-NaOH, PH 8.5, 100 mM KCl, 10 mM DTT, 0.01% bovine serum albumin), 0.04 µM linear DNA substrates, and 0.1 µM phiC31 integrase. Reactions were performed at 30°C for 30 min and then stopped at 75°C for 10 min. Subsequently, reaction products were directly separated on a 1% agarose gel and stained with ethidium bromaide (1 µg/ml) for 20 min. A 1.4 Kb linear *attR* fragments band densities were analyzed by using a UVP-GDS8000 gel analysis system (Ultra-Violet Products Ltd, Cambridge, UK). To measure the residual activities more precisely, the reactions were performed with higher concentraions of the proteins,and the activities relative to that of wild-type phiC31 integrase were calculated.

### Recombination Assay *in E. coli.*

Recombination assays *in vivo* were performed as described previously [38]. A 200 ng sample of pET22b-MC31 plasmid and a 100 ng sample of the pBCPB+ plasmid were co-transformed into Top10 bacteria, which were allowed to recover for 1 h, then spread on agar plates containing 25 µg/ml chloramphenicol, 50 µg/ml ampicillin, and 50 µg/ml 5-bromo-4-chloro-3-indolyl b-Dgalactoside (X-Gal), and grown at 37°C. If an intramolecular integration event occurs, the *lacZ* gene located between the *attB* and *attP* sites will be excised, and a resulting colony will be white. The frequency of intramolecular recombination was calculated as the number of white colonies divided by the total number of colonies and multiplying by 100. Each frequency is derived from at least three independent experiments.

DNA isolated by plasmid extraction from five different white colonies was subjected to sequence analysis for further verifying the recombination. The primers *attB*-F (5′-GGCGAGAAAGGAAGGGAAGA-3′) and *attP*-R (5′- ATTAACCCTCACTAAAGGGA-3′) were used to specifically amplify the wild type attL junction in integrase-reacted pBCPB.

### DNA Binding Assays

DNA fragments containing the attachment sites *attB* and *attP* were prepared for use as substrates in EMSA [28], [42]. Groth et al demonstrated that these att sites are functional with the minimal lengths of 34 bp for *attB* and 39 bp for *attP* [22]. In our experiments, the *attB* and *attP* fragments used as substrates are a 41 bp and a 50 bp in length, respectively. Linear substrates containing the *attB* or *attP* sequence were synthesized and labeled by Invitrogen Company, Shanghai, China. The sequences of biotin- DNA probes that were double-strand end-labeled were *attB* (5′- CGGGTGCCAGGGCGTGCCCTTGGGCTCCCCGGGCGCGTACT-3′ and 5′- AGTACGCGCCCGGGGAGCCCAAGGGCACGCCCTGGCACCCG-3′) and *attP*(5′-GTAGTGCCCCAACTGGGGTAACCTTTGAGTTCTCTCAGTTGGGGGCGTAG-3′ and 5′-CTACGCCCCCAACTGAGAGAACTCAAAGGTTACCCCAGTTGGGGCACTAC-3′). PhiC31 DNA binding activity was detected by using a LightShift Chemiluminescent EMSA kit (Pierce, USA) according to the manufacturer's instructions. Briefly, the wild type and mutant protein were mixed with binding buffer (50 ng/µl Poly, 0.05% NP-40, 50 mmol/L KCl, 5 mmol/L MgCl_2_ and 10 mmol/L ethylenediamine tetraacetic acid) in a total volume of 20 µL containing 0.4 ng the biotin- *attB* DNA probes or biotin- *attP* DNA probes, respectively. For competition experiments, usually 100-fold excess unlabeled competitor DNA *attB/attP* was added to the reaction mixture on ice for 10 min prior to the addition of the labeled probe. The mixtures were incubated for 30 min at room temperature before the addition of 3 µL of 5×loading buffer. Samples were loaded onto a native 8% polyacrylamide gel, and were electrophoresed at 100 V until the bromophenol blue dye had migrated approximately two-thirds to three-quarters of the length of the gel, and then the binding reactions were electro blotted onto nylon membrane at 380 mA (∼100 V) for 1 h. The membrane was cross-linked for 2 min at a distance of approximately 0.5 cm from an ultraviolet lamp equipped with 254 nm bulbs, and biotin-labeled DNA was visualized by using enhanced chemoluminescence. The images were developed on X-ray film and the band densities were analyzed by using a UVP-GDS8000 gel analysis system (Ultra-Violet Products Ltd, Cambridge, UK). The amount of free DNA substrate was plotted against concentrations of integrase to calculate the apparent binding affinities, Kd.

### DNA Synapsis and Cleavage Assays

DNA synapsis and cleavage assays were performed as described previously [28]. For synapsis assays, the unlabelled ‘partner’ fragments *attP* (198 bp) prepared by PCR amplification from pTA-*attP* with primers *attP*-F(5′- ACTGACGGACACACCGAAGC-3′) and *attP*-R (5′- TCGTAAGCACCCGCGTACGTGTC-3′). 0. 4 ng labelled probe *attB* (41 bp) was mixed with 12 ng of cold partner fragment and 0.8 µg integrase in 20 µL binding buffer. Reactions were incubated at 30°C for 2 h prior to electrophoresis. To detect the cleavage of the DNA fragments by integrase, samples required proteinase treatment. Briefly, samples were first heat inactivated by incubation at 72°C for 10 min and then treated proteinase K (1 µg in 5 mM EDTA, 0.5% SDS; incubated at 50°C for 30 min). Finally, samples were incubated at 72°C for an additional 10 min to inactivate the proteinase prior to polyacrylamide gel electrophoresis (5% polyacrylamide, 1x Tris-borate-EDTA).

### FT-IR Spectroscopy and Circular Dichroism Spectroscopy

FT-IR measurements of the phiC31 integrase in heavy water were performed as described previously [48]. Circular dichroism measurements were performed on a Jasco 715 spectropolarimeter (Jasco, Tokyo, Japan) using a quartz cuvette with 0.1 cm path length over a wavelength range of 190–250 nm at 25°C. Protein was dissolved in 0.15 M sodium chloride, pH 7.0. The CD data were collected every 0.2 nm and were the average of four scans. The Results were expressed in units of molar ellipticity per residue (deg cm2 dmol-1) and plotted versus the wavelength. The bandwidth was set at 1.0 nm and the sensitivity at 25 mdeg. The response time was 0.5 s. In all cases, baseline scans of control buffers were subtracted from the experimental readings. CD data were processed by Jasco secondary structure estimation software.

### Computer Analysis

For sequence analysis we used the Align X in VNTI 9.0 software package. Protein secondary structure predictions were made by using the servers PROF (http://www.aber.ac.uk/~phiwww/prof/) and Jpred 3 (http://www.compbio.dundee.ac.uk/~www-jpred/).
